# CA IX Stabilizes Intracellular pH to Maintain Metabolic Reprogramming and Proliferation in Hypoxia

**DOI:** 10.3389/fonc.2020.01462

**Published:** 2020-09-02

**Authors:** Martin Benej, Eliska Svastova, Radivojka Banova, Juraj Kopacek, Adriana Gibadulinova, Martin Kery, Simona Arena, Andrea Scaloni, Monica Vitale, Nicola Zambrano, Ioanna Papandreou, Nicholas C. Denko, Silvia Pastorekova

**Affiliations:** ^1^The Ohio State University Wexner Medical Center and OSU Comprehensive Cancer Center, Columbus, OH, United States; ^2^Department of Tumor Biology, Institute of Virology, Biomedical Research Center, Slovak Academy of Sciences, Bratislava, Slovakia; ^3^Proteomics and Mass Spectrometry Laboratory, ISPAAM, National Research Council, Naples, Italy; ^4^Dipartimento di Medicina Molecolare e Biotecnologie Mediche, Università degli Studi di Napoli Federico II, Naples, Italy; ^5^CEINGE Biotecnologie Avanzate, Naples, Italy

**Keywords:** carbonic anhydrase IX, hypoxia, pH regulation, glycolysis, metabolism

## Abstract

Tumor hypoxia represents a severe microenvironmental stress that is frequently associated with acidosis. Cancer cells respond to these stresses with changes in gene expression that promote survival at least in part through pH regulation and metabolic reprogramming. Hypoxia-induced carbonic anhydrase IX (CA IX) plays a critical adaptive role in response to hypoxic and acidic environments by catalytically hydrating extracellular CO_2_ to produce bicarbonate for buffering intracellular pH (pHi). We used proteome-wide profiling to study the cellular response to transient CA IX knockdown in hypoxia and found a decrease in the levels of key glycolytic enzymes and lactate dehydrogenase A (LDHA). Interestingly, the activity of LDH was also decreased as demonstrated by native in-gel activity assay. These changes led to a significant reduction in glycolytic flux and extracellular lactate levels in cancer cells *in vitro*, contributing to a decrease in proliferation. Interestingly, addition of the alternative LDH substrate alpha-ketobutyrate restored LDHA activity, extracellular acidification, pHi, and cellular proliferation. These results indicate that in the absence of CA IX, reduction of pHi disrupts LDHA activity and hinders the cellular capacity to regenerate NAD^+^ and secrete protons to the extracellular space. Hypoxia-induced CA IX therefore mediates adaptation to microenvironmental hypoxia and acidosis directly, by enzymatically converting extracellular CO_2_ to bicarbonate, and indirectly, by maintaining glycolysis-permissive intracellular milieu.

## Introduction

The physiological stresses that exist in the tumor microenvironment are critical selective factors in tumorigenesis that drive the behavior of cancer cells' proliferation, invasiveness, resistance to therapy, and survival (1). Tumor blood vessels are often poorly formed and therefore fail to deliver sufficient oxygen and nutrients, and to remove resulting waste products from metabolically active tumor cells (2). Low oxygen tension initiates an adaptive response mediated largely by stabilization of hypoxia-inducible factor 1α (HIF-1α) (3). Activation of the HIF-1 pathway triggers the expression of dozens of adaptive target genes that contribute to an aggressive tumor phenotype (2).

One of the most significant adaptive responses to hypoxia is the shift of glucose metabolism from mitochondrial oxidative phosphorylation (OXPHOS) to glycolysis due to induction of glycolytic genes and downregulation of mitochondrial function by pyruvate dehydrogenase kinase 1 (PDHK1) (4). While glycolysis generates only two molecules of ATP and two molecules of NADH per molecule of glucose converted to pyruvate, it can function efficiently in reduced oxygen, generating energy and providing intermediates for *de novo* synthesis of amino acids, nucleotides, and lipids to support cellular proliferation (5, 6). In hypoxia, PDHK1 expression reduces pyruvate dehydrogenase (PDH) activity and flow of pyruvate into TCA reactions so that pyruvate generated from glycolysis is predominantly converted into lactate through a process catalyzed by lactate dehydrogenase (LDH) (7). Enhanced glycolytic metabolism causes increased production of lactate, protons and carbon dioxide, resulting in microenvironmental acidosis (8). Maintaining physiological intracellular pH (pHi) in these conditions is essential for cell function (9). Cancer cells respond by upregulating key pH regulatory systems including Na^+^/H^+^ exchangers (NHEs), vacuolar (V-type) H^+^ ATPases, monocarboxylate and bicarbonate transporters as well as the plasma membrane carbonic anhydrase IX (CA IX) (8). CA IX is a HIF1-responsive Zn^2+^-metalloenzyme that catalyzes reversible hydration of extracellular CO_2_ to bicarbonate ion and proton, and cooperates with bicarbonate transporters in bicarbonate import. This process provides bicarbonate ions for neutralization of the pHi and simultaneously extrudes protons for extracellular pH (pHe) acidification (10, 11). Moreover, CA IX is an active player in tumor cell adhesion-migration-invasion, and, due to its association with aggressive cancers, it can serve as a diagnostic/prognostic biomarker and therapeutic target (12).

The aim of this study was to investigate the complexities of CA IX-mediated adaptive response to hypoxia in cancer cell biology using an unbiased proteomic approach. Here we report our findings from proteome-wide profiling of hypoxic cells to identify proteins responsive to reduced CA IX expression. We demonstrate that reduction of CA IX levels decreases the abundance of key glycolytic enzymes. Functional analysis showed that CA IX loss decreases both level and activity of lactate dehydrogenase A, exacerbating the drop in pHi value and reducing glycolytic rate. We also show that addition of the alpha-ketobutyrate (α-KB), a four carbon metabolite that can be utilized by LDH to regenerate NAD^+^ in pyruvate deficiency (13), compensates for low pyruvate levels generated by decreased glycolytic flux. Moreover, α-KB addition also restores extracellular acidification rate (ECAR), pHi and proliferation in CA IX-deficient cells. These results indicate that in the absence of CA IX, reduction of pyruvate flux and lactate dehydrogenase activity further compromises the cellular capacity to secrete protons to the extracellular space and maintain pHi.

## Materials and Methods

### Cell Culture

HeLa (cervical cancer, RRID:CVCL_0030), HT-1080 (fibrosarcoma, RRID:CVCL_0317), A-549 (lung cancer, RRID:CVCL_0023), and C-33A (cervical cancer, RRID:CVCL_1094) cancer cell lines were obtained from American Type Culture Collection (ATCC). Stable doxycycline-inducible HeLa CA IX knockdown (HeLa DOX) cells were generated as previously described (14). Transient HeLa (HeLa KD), A-549 (A-549 KD), and HT-1080 (HT-1080 KD) CA IX knockdown cells were produced by direct transfection of a pool of CA9 siRNA oligonucleotides (Dharmacon, GE Healthcare); for sequences see Supplementary Methods. Stable HT-1080 CA IX knockout cells (HT-1080 KO) were generated using CRISPR/Cas9-mediated genome editing, for a detailed protocol and a table with all cell lines and their modifications see Supplementary Methods. Stable dominantly negative HeLa CA IX-dCA deletion variants lacking the catalytic domain (HeLa dCA) were generated as previously described (12). C-33A cells that do not express endogenous CA IX were stably transfected with the full-length CA IX-encoding pcDNA3.1 plasmid to generate C-33A-FL cells as described previously (12). All cell lines but C-33A are tumorigenic, express CA IX in response to hypoxia, and CA IX suppression/deletion reduces their tumor-forming capacity *in vivo*. Cells were grown in Dulbecco's Modified Eagle's Medium (DMEM) containing 25 mM D-glucose, 4 mM glutamine and 44 mM sodium bicarbonate in 5% CO_2_, if not stated otherwise. The cells were exposed to hypoxia (2 and 1% O_2_) in Hypoxia workstation (Ruskinn Technologies) and H35 Hypoxic Workstation (Hypoxygen) with 5% CO_2_ at 37°C and concurrent 0.5% serum starvation, if not stated otherwise. In some experiments, we used a hypoxia mimetic DMOG as described. Cell counts and cellular proliferation were established using hemocytometer to determine the cell number and trypan blue exclusion assay to establish the fraction of viable/dead cells. Normalized values were plotted with respect to each control so that multiple experiments could be averaged together for comparison purposes. All human cell lines have been authenticated using STR profiling within the past 3 years. All experiments were performed with mycoplasma-free cells.

### 2D Proteomic Profiling

HeLa DOX cells were incubated for 48 h in DMEM media containing 1 mM dimethyloxalyglycine (DMOG) and reduced serum (0.5% fetal calf serum). CA IX knockdown was induced by addition of 250 pg doxycycline (DOX) into growth media 72 h prior to seeding. Control and DOX-treated HeLa DOX cells were labeled with Cy3 and Cy5, respectively; their 1:1 mix was labeled with Cy2, and used as an internal standard for each replicate analytical gel (*n* = 3). Significantly altered protein spots of interest were identified using DeCyder software (GE Healthcare) and matched with their counterparts on SYPRO Ruby-stained preparative gel, from which they were picked and analyzed for protein identification by nLC-ESI-LIT-MS/MS. The over/underrepresentation cutoff was +- 1.38. See Supplementary Methods for a detailed protocol.

### Immunoblotting

Proteins were extracted with 1% v/v Triton X-100, 0.5% w/v Nonidet NP-40, 150 mM NaCl, 50 mM Tris, pH 7.5, quantified by BCA Kit (Thermo Scientific) and separated on 10% SDS-PAGE under reducing conditions. Proteins of interest were detected using the antibodies directed toward CA IX (10, 12), ALDOA (Abcam), PGK1 (Abcam), ENO1 (Abcam), LDH (Abcam), LDHA (Cell Signaling), pTyr10 LDHA (Cell Signaling), and β-actin (Santa Cruz Biotech). All replicates of the individual experiments were analyzed from the respective run with appropriate loading controls. Where similar Mw precluded detection of all proteins on the same membrane, replicate membranes were used.

### Intracellular pH Measurements

For pHi measurements, cells were incubated for 48 h in 1% hypoxia. On the day of the assay, cells were washed with Hank's Balanced Salt Solution before loading with 7 μmol/l SNARF-1 (5-(and) 6-Carboxy SNARF-1 acetoxymethyl ester, Thermo Fisher) for 30 min, at 37°C. pHi was calculated from a calibration curve with 10 μmol/l nigericin (Sigma) by determining the 640/580 nm emission wavelength ratios according to the manufacturer's instructions.

### Lactate Assay

Growth media from individual experiments were collected and immediately frozen at −80 °C. Extracellular lactate concentrations in the growth media were investigated using Lactate Assay Kit (Sigma Aldrich) according to the manufacturer's protocol. The principle of the assay is based on reaction in which lactate dehydrogenase catalyzes oxidation of lactate and the formed NADH interacts with reagent solution to give a colorimetric readout proportional to extracellular lactate concentration in the media. Samples were analyzed in triplicates and the resulting concentrations were determined from the calibration curve. Normalized values were plotted with respect to each control so that replicate experiments could be averaged together for comparison purposes. Absolute mean concentrations are included in the figure legend.

### LDH Activity In-Gel Assay

LDH isoforms in whole cell native PAGE lysate samples were separated on a native 5% PAGE gel in 25 mM tris and 250 mM glycine, pH 9.5. Native gel electrophoresis was conducted at 100 V in 5 mM Tris, 40 mM Glycine, pH 9.5 running buffer. Once the isoforms were separated, the gel was washed in 0.1M Tris, pH 8.5 and incubated in LDH reaction solution containing 3.24 mg/ml lactate, 0.3 mg/ml nicotinamide adenine dinucleotide (NAD), 0.8 mg/ml nitroblue tetrazolium and 0.167 mg/ml phenazine methosulfate dissolved in 0.01 M Tris, pH 8.5. The colorimetric reaction is proportional to LDH activity and the electromobility shift is specific for LDH3-5 isoforms based on their LDHA:LDHB subunit combination.

### Analysis of Extracellular Acidification and Oxygen Consumption Rates

Measurements of extracellular acidification rate (ECAR) and oxygen consumption rate (OCR) were performed using the Seahorse XF96 analyzer (Agilent). Briefly, 1 × 10^4^ cells per well were seeded in a 96-well-XF cell culture microplate in DMEM with 10% FCS and exposed to hypoxia for 24 h. The assays were then performed in bicarbonate and serum-free DMEM medium following a 2-h degassing step. After establishing baseline levels of ECAR, cells were treated with 1 μg/ml oligomycin A to inhibit mitochondrial ATP-synthase and with 25 mM 2-deoxy-D-glucose to inhibit glycolysis. In the experiment with CA inhibitor, ECAR data was transformed post-run to proton production rate (PPR) that reflects the number of protons extruded on a linear rather than the logarithmic pH scale.

### Statistics

Data are presented as mean values ± standard deviation. Student's t-test was used for calculation of significance in differences. In the figures shown, a significance level of *p* ≤ 0.05 is marked with ^*^, and *p* ≤ 0.01 with ^**^.

## Results

### CA IX Knockdown Decreases the Abundance of Key Glycolytic Enzymes

**Figure 1:**
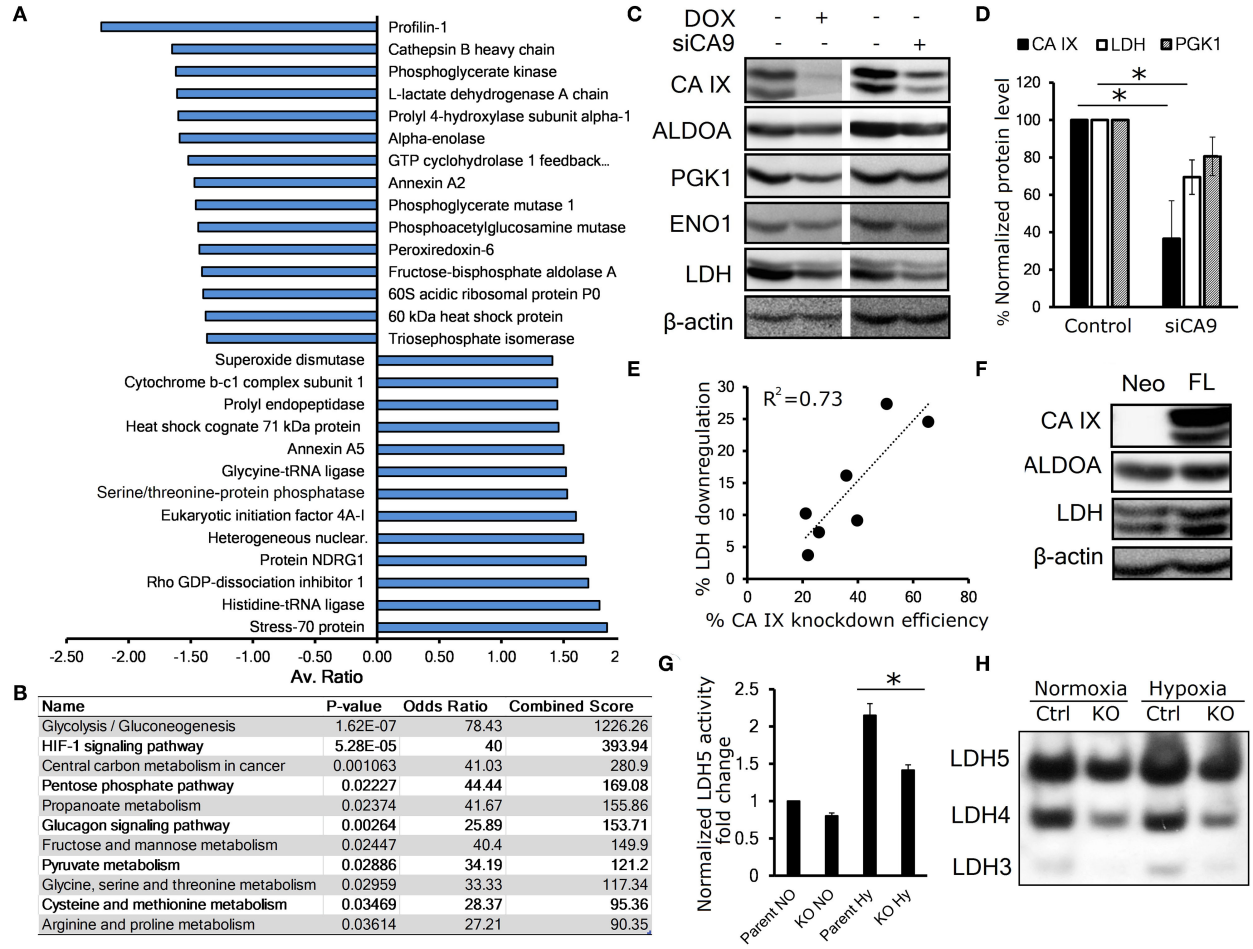
CA IX knockdown decreases the abundance of key glycolytic enzymes. **(A)** Average fold change of proteins affected by doxycycline (DOX)-induced CA IX knockdown in hypoxic HeLa DOX cells incubated in 0.5% serum for 48 h, determined by 2D-DIGE analysis (*n* = 3). **(B)** KEGG pathway analysis of under-represented proteins in response to knockdown of CA IX. *P*-value is computed from the Fisher exact test for probability of the set of genes belonging to a specific pathway. Odds ratio and Combined score are additional biostatistical means representing the probability that the selected gene set is component of the respective pathway. All computations were provided by the Enrichr data analysis platform (15, 16). **(C)** Representative immunoblots showing the effect of CA IX knockdown in HeLa DOX (left) and HeLa KD (right) cells incubated in 2% O_2_ for 48 h (*n* = 3). **(D)** Impact of CA IX knockdown (siCA9) on LDH and PGK1 levels normalized to β-actin in HeLa KD cells incubated in 2% O_2_ for 48 h (*n* = 5). **(E)** Dose-response relationship between CA IX knockdown and reduction of LDH levels in hypoxic HeLa KD cells. **(F)** Representative immunoblot showing increased ALDOA and LDH in CA IX-transfected C-33 A cells (C33 A-FL) that lack endogenous CA IX expression incubated in 2% O_2_ for 48h (*n* = 3). **(G)** LDH5 activity of HT-1080 CA IX knockout (HT-1080 KO) in normoxia and 48h 1% hypoxia (*n* = 3). **(H)** Representative LDH activity assay showing separated LDH5-LDH3 isoforms stained for LDH activity (*n* = 3). Error bars are ±SD. *P*-values were calculated against control by t-test. *P < 0.05.

To investigate the effect of CA IX knockdown on the whole proteome of hypoxic HeLa cells, we generated HeLa DOX, a stable cell line with doxycycline (DOX)-inducible short hairpin-mediated CA IX knockdown and performed two-dimensional differential in-gel electrophoresis (2D-DIGE) combined with nano-liquid chromatography-electrospray linear ion trap tandem mass spectrometry (nLC-ESI-LIT-MS/MS). These experiments identified 35 gel spots with significantly altered abundance, 14 of which were over-represented and 21 were under-represented after CA IX knockdown (Figure 1A, Figures S1A,B, S2A, Table S1). These altered spots were associated with 28 proteins involved primarily in glycolytic metabolism, but also in cell cytoskeleton and transport, and other metabolic pathways known to be involved in cancer progression and stress response (Figure S1B). KEGG Pathway analysis of the under-represented dataset revealed significant impact of CA IX suppression on the glycolytic/gluconeogenetic pathway (Figure 1B). The most striking effect was observed in relationship to glycolytic metabolism, where key glycolytic enzymes displayed a decreased abundance. Considering the importance of glycolysis in adaptation of cancer cells to hypoxia, we decided to further evaluate a possible link of CA IX to the control of glycolysis.

We first validated the proteomic results by immunoblotting analysis of HeLa DOX cells exposed for 48 h to 1% hypoxia with concurrent serum starvation. To rule out the possibility that the downregulation was due to the effect of doxycycline, we also analyzed HeLa KD cells with transient CA IX siRNA knockdown (siCA9). In both cases, suppression of CA IX led to decreased levels of fructose-bisphosphate aldolase A (ALDOA), phosphoglycerate kinase 1 (PGK1), enolase (ENO1), and most significantly lactate dehydrogenase (LDH) (Figures 1C,D). Interestingly, we observed a dose-response relationship between CA IX and LDH levels, suggesting a functional interplay of these proteins (Figure 1E). LDH level was reduced also in hypoxic HT-1080 KO cells with CRISR/Cas9-mediated knock-out of CA9 gene, in comparison to control HT-1080 cells (Figure S2B). The converse of these findings was confirmed with overexpression of full-length CA IX in C-33A cervical cancer cells (C-33A-FL), that lack endogenous CA IX expression, where we found increased LDH levels (Figure 1F).

These observations are consistent with the proteomic findings and established our interest in LDH as a key enzyme in glucose metabolism that is most significantly impacted by CA IX knockdown. LDH is a tetramer comprising four subunits encoded either by LDHA or LDHB genes and based on the subunit LDHA:LDHB composition, there are five known LDH isozymes, LDH1-5 (17). Hypoxia-induced LDHA has a high affinity to pyruvate, thus preferentially converting it to lactate, while LDHB favors the reverse reaction (18). In order to analyze the effect of CA IX suppression on LDH activity, we used an in-gel enzyme assay based on separation of LDH isoforms with differential mobility in native PAGE gel followed by staining with the substrate-containing reaction solution (11). Out of five LDH isoforms, HT-1080 cells displayed isoforms 5-3 (LDH5 being A subunit homotetramer, LDH4 being A3B1, and LDH3 being A2B2 heterotetramers). LDH2 and LDH1 were not detected due to low expression of LDHB. We found increased LDH activity of all three isoforms in HT-1080 cells exposed to hypoxia compared to normoxia, whereas a significant reduction of LDH activity was evident in CA IX-deficient HT-1080 KO cells compared to controls both in normoxia and hypoxia. These results suggest that CA IX suppression affects both level and activity of LDHA. (Figures 1G,H, Figure S2C).

### Catalytic Activity of CA IX Maintains Glycolytic Flux

**Figure 2:**
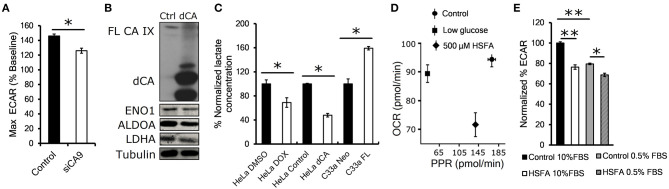
Catalytic activity of CA IX maintains glycolytic flux. **(A)** Maximum glycolytic flux rate of HT-1080 KD cells incubated for 24 h in 1% O_2_, treated with oligomycin A after establishing baseline ECAR (extracellular acidification rate). **(B)** Representative western blot showing decreased levels of glycolytic enzymes ENO1, ALDOA and LDHA in HeLA dCA cells that overexpress CA IX lacking the catalytic domain (dCA, right lane). HeLa dCA cells were incubated in 2% O_2_ for 48 h (*n* = 3), the full length CA IX (FL CA IX) is shown in the top of the blot **(C)** Normalized extracellular lactate concentration of HeLa DOX, HeLa dCA, and C-33A-FL cells incubated in 2% O_2_ for 48 h. Mean baseline lactate concentrations are: Hela DMSO 0.11 mM, HeLa DOX 0.08 mM, HeLa Control 0.09 mM, HeLa dCA 0.04 mM, C-33A-Neo 0.16 mM, C-33A-FL 0.26 mM. **(D)** Seahorse analysis of oxygen consumption rate (OCR) and proton production rate (PPR) in posthypoxic parental HT-1080 cells in low glucose (1mM) or with CA IX inhibitor 4-aminometylbenzensulfonamid (HSFA). **(E)** Normalized ECAR in HT-1080 cells pre-incubated in 1% O_2_ for 24 h in 10 and 0.5% serum. After establishing the baseline, the cells were treated with HSFA for 30 min (right). Error bars are ±SD. *P*-values were calculated against Control by t-test. *P < 0.05; **P < 0.01.

Proteomic results suggested that CA IX knockdown significantly affects the glycolytic pathway. Therefore, in the next step we examined the functional impact of transient CA IX knockdown on glycolysis and lactate production using Seahorse XF analysis of HT-1080 cells, which exhibit increased glycolytic flux due to heterozygous IDH1 mutation (19). We observed a significant reduction of the maximal glycolytic capacity in HT-1080 cells with transient CA IX knockdown (HT-1080 KD) (Figure 2A). In order to examine the necessity of catalytic activity of CA IX on glycolytic flux and lactate production, we generated HeLa dCA cells that express a dominant inhibitory catalytically inactive CA IX. Immunoblot analysis of extracts from these cells after exposure to hypoxia and low serum indicated a significant reduction of expression of ENO1, ALDOA, and LDHA, suggesting that CA IX activity is essential for control of cellular metabolism in hypoxia (Figure 2B). Because lactate is a major end-product of glycolysis in hypoxia, we directly measured extracellular lactate concentration in the growth media of HeLa DOX, HeLa dCA or C-33A-FL cells. Reduction of CA IX activity by either knockdown or expression of the catalytically inactive dCA protein caused a significant reduction of lactate concentration, while C-33A-FL cells with ectopic CA IX expression showed the opposite effect (Figure 2C). This model relating CA IX activity to glycolytic rate was further supported by Seahorse analysis of posthypoxic HT-1080 cells treated with homosulfanilamide (HSFA), a CA IX-selective catalytic inhibitor (10). Figure 2D shows that treatment with HSFA reduced proton production rate (PPR) relative to oxygen consumption rate (OCR). Further analysis of these cells indicated that HSFA reduces ECAR equally well after hypoxic growth in either high or low serum conditions (Figure 2E). Collectively, these findings show that reduction of CA IX catalytic activity by genetic manipulation or its pharmacological inhibition significantly reduce the glycolytic flux.

### Alternative LDH Substrate Alpha-Ketobutyrate Restores Perturbed Proliferation and pHi in CA IX Deficient Cells

**Figure 3:**
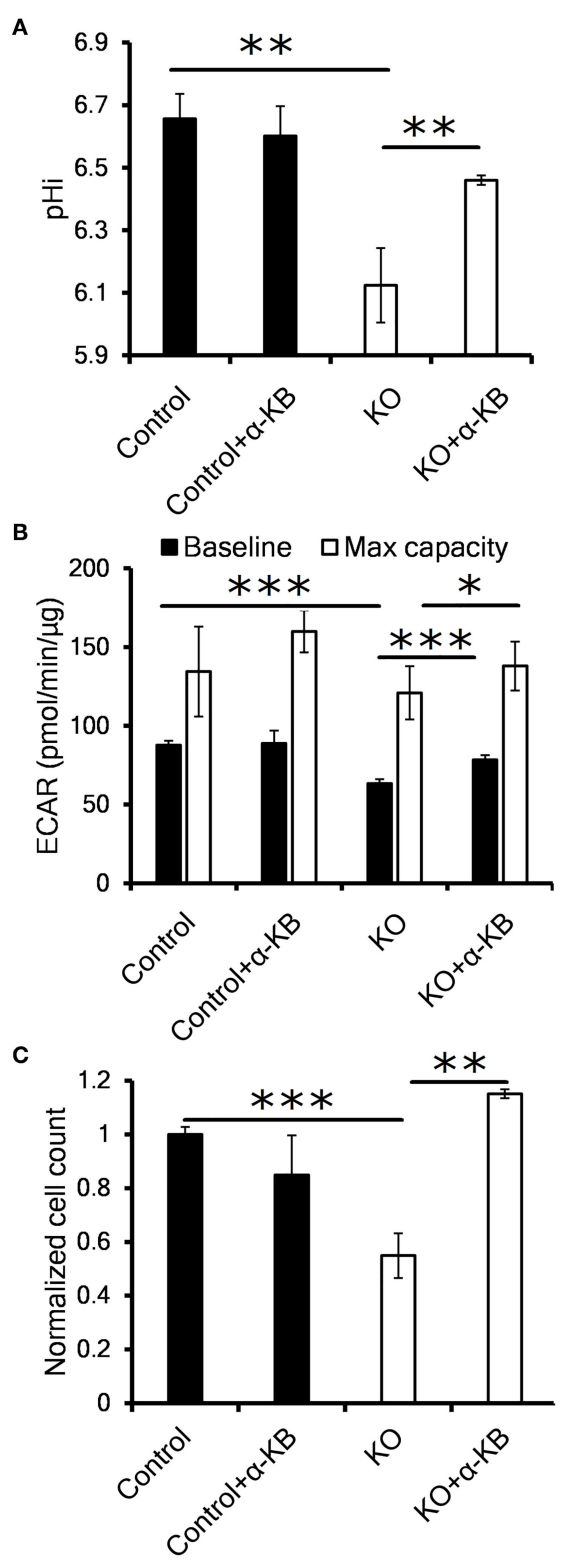
Alternative LDH substrate alpha-ketobutyrate restores pHi, proliferation and LDH activity in CA IX deficient cells. **(A)** Intracellular pH (pHi) of HT-1080 KO cells incubated for 48 h in 1% O_2_ with 1 mM alpha-ketobutyrate (α-KB). **(B)** Seahorse analysis of baseline ECAR (black) and the maximal glycolytic capacity (white) of HT-1080 KO cells. **(C)** Normalized cell number of HT-1080 KO cells after 72 h in 1% O_2_ in the presence or absence of α-KB. Error bars are ±SEM. *P*-values were calculated against control by *t*-test. *P < 0.05; **P < 0.01 ***P < 0.001.

Previous reports have shown that suppression or inhibition of CA IX disrupts pH control in hypoxia and reduces cellular proliferation and/or tumor growth (20–22). In order to investigate the mechanism of reduced proliferation in the context of our observation that CA IX suppression decreases the glycolytic flux, we tested several compounds that modify different aspects of glucose metabolism to see if they could restore the growth of CA IX deficient cells in hypoxia. Inhibition of LDH (oxamate), or pyruvate entry into the mitochondria (UK5099), or *de novo* NAD synthesis (FK866) did not restore the growth of HT-1080 KO cells (Figure S2D). However, addition of the alternative LDH substrate alpha-ketobutyrate (α-KB) specifically recovered the hypoxic proliferation of HT-1080 KO cells (Figure 3). The same effect of α-KB was observed in A-549 KD cells with transient CA IX knockdown (Figure S2E).

In order to clarify a regulatory link between CA IX and LDH implicated in sustained cell proliferation, we analyzed the effect of α-KB on intracellular pH (pHi). It is well-known that intracellular acidosis inhibits glycolysis and subsequently perturbs cell proliferation and that CA IX is a key component of pHi regulatory machinery protecting cells from intracellular acidosis. Using the pH sensitive dye SNARF-1, we first confirmed that CA IX loss resulted in reduced pHi after HT-1080 and A-549 cells' exposure to hypoxia for 48 h in 0.5% serum (Figure 3A, Figure S2F). Interestingly, addition of α-KB reversed the drop in pHi in hypoxic HT-1080 KO cells as well as in A-549 KD cells with transient CA IX knockdown, but had no effect on pHi in CA IX positive cultures.

We next used Seahorse XF assay to determine if the reduced pHi was the result of proton secretion to the extracellular space (i.e. ECAR). In similarly treated cells, we confirmed significant decrease of both the baseline and maximal ECAR of HT-1080 KO and A-549 KD cells (Figure 3B, Figure S2G). Addition of α-KB increased proton secretion in CA IX deficient cells, in agreement with the increased pHi seen in Figure 3A. Moreover, α-KB could restore the diminished hypoxic proliferation of HT-1080 KO and A-549 KD cells without affecting proliferation of CA IX positive cells (Figure 3C, Figure S2E). These findings support a model in which intracellular acidosis as a consequence of CA IX suppression reduces LDHA level/activity, the glycolytic flux and ultimately cellular proliferation and that the pHi-increasing function of α-KB can reverse all these effects.

### Intracellular Acidosis Decreases LDHA Level and Activity

**Figure 4:**
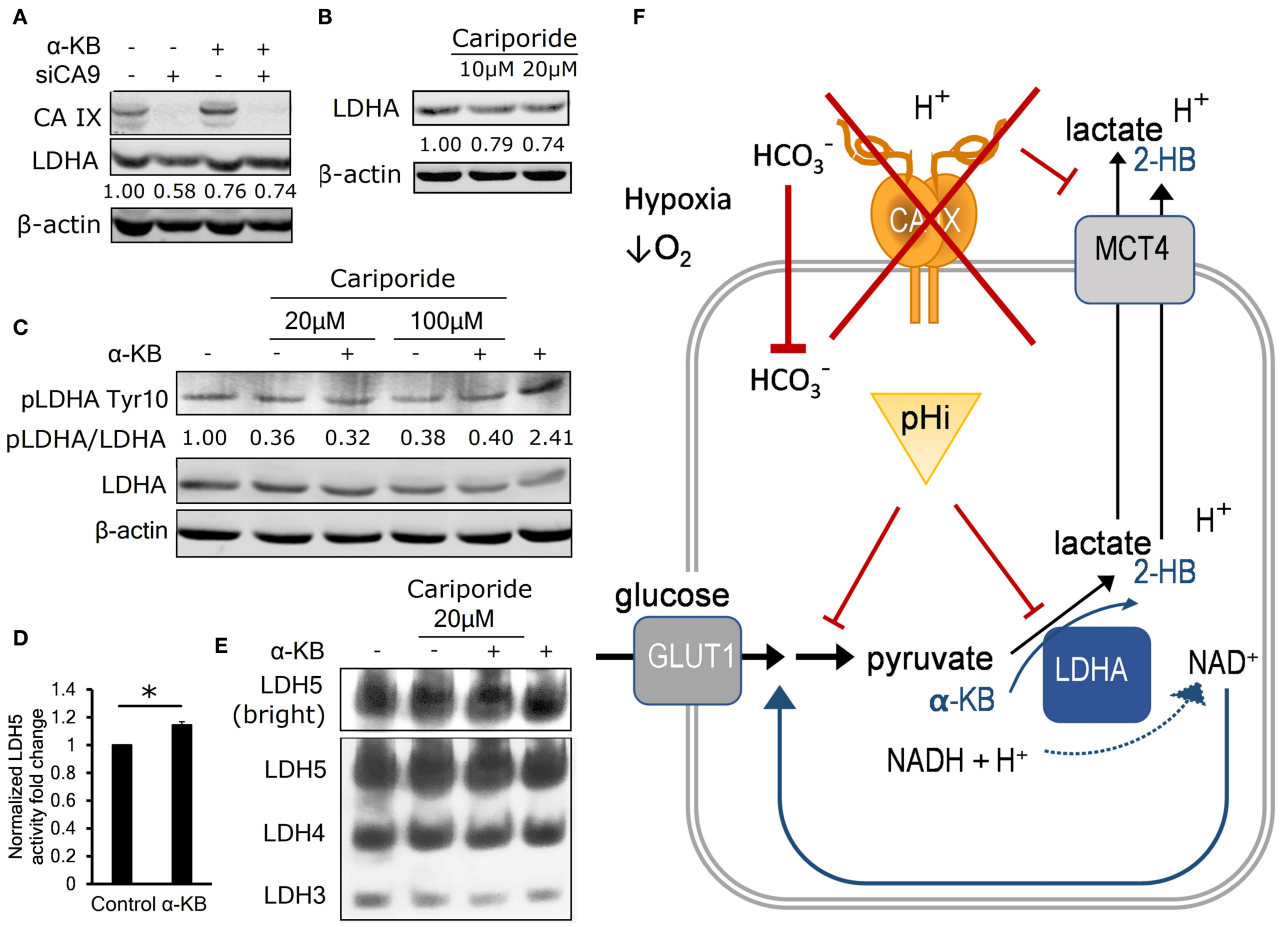
Intracellular acidosis decreases LDHA level and activity. **(A)** Representative immunoblot (*n* = 3) showing LDHA levels in control and α-KB-treated A-549 KD cells incubated for 48 h in 1% O_2_, numbers represent relative LDHA levels quantified against β-actin in the presented blot. **(B)** Representative immunoblot (*n* = 3) showing the effect of sodium-hydrogen antiporter 1 (NHE-1) inhibition by cariporide on LDHA levels in normoxic parental HT-1080 cells, numbers represent relative LDHA levels quantified against β-actin in the presented blot. **(C)** Representative immunoblot (*n* = 3) showing the effect of 48h cariporide and α-KB treatment on the levels of activating LDHA tyrosine 10 phosphorylation (pLDHA Tyr10) in normoxic HT-1080 cells, numbers represent pTyr10/LDHA ratio in the presented blot. **(D)** LDH5 activity of normoxic HT-1080 cells in response to α-KB (*n* = 3). **(E)** Representative LDH activity assay showing LDH5 in the enhanced contrast field (bright, top) and resolved LDH5-LDH3 isoforms (bottom) in response to α-KB and 20 μM cariporide. **(F)** Schematic indicating the interaction between CAIX, pHi, and the regulation of glycolysis and lactate production. Error bars are ±SEM. *P*-values were calculated against Control by *t*-test. *P < 0.05; **P < 0.01 ***P < 0.001.

If decreased pHi resulted in the reduction in LDHA level and activity, then α-KB should also recover LDHA protein levels in cells with CA IX suppression. Consistent with this expectation, immunoblot analysis of hypoxic A-549 KD and HeLa KD cells showed restored LDHA levels after addition of α-KB (Figure 4A, Figures S2H,I).

To find out whether acidic pHi can lead to reduced LDHA protein levels independently of hypoxia and CA IX, we inhibited a sodium-hydrogen exchanger NHE1, which is another important pH regulator previously shown to support glycolysis and malignant phenotype (23). We used the NHE1 inhibitor cariporide to treat normoxic HT-1080 cells for 24 h and observed that pHi decreased by 0.4 pH units (Figure S2J), similarly treated cells showed a 25% decrease of LDHA protein abundance by immunoblot (Figure 4B, Figure S2K). These results indicate that LDHA level is decreased by reduced pHi, without involvement of hypoxia and/or CA IX, suggesting that its expression or stability is regulated by mechanisms downstream of reduced intracellular pH.

We then investigated whether LDHA activity is regulated by intracellular signaling in pHi-dependent manner via analysis of the phosphorylation status of Tyr10 residue on LDHA, which can be modified by several receptor and non-receptor tyrosine kinases (24). pTyr10 is thought to enhance LDHA activity by actively supporting the formation of the LDHA tetramer LDH5 and enhancing NADH binding (24). We observed a 60% reduction of the ratio of pTyr10/LDHA in normoxic HT-1080 cells in response to cariporide treatment indicating that acidic pHi reduces LDHA activity along with its level (Figure 4C). In addition, both the LDH5 formation and the LDHA protein level were elevated in response to α-KB treatment (Figures 4D,E, Figure S2L), consistently with its ability to substitute for LDH substrate and regenerate NAD^+^ formation to feed the glycolytic flux. This finding further reinforces the view that pHi changes influence both LDHA abundance and activity irrespective of hypoxia.

These results support a model, in which CA IX suppression causes a compound effect on pHi: by first decreasing the cell's buffering capacity that reduces pHi, which in turn reduces LDHA activity that compromises the cell's ability to sustain glycolysis and secrete lactate. Addition of α-KB reverses these effects by two actions. First, in cells with decreased glycolytic flux, conversion of α-KB to 2-hydroxybutyrate by LDHA compensates for pyruvate deficiency to regenerate NAD^+^ and stimulate secretion of protons (i.e., ECAR). Second, LDHA mediated conversion of α-KB to 2-HB increases LDH activity by stimulating pTyr10. These actions of α-KB combine to increase pHi, which in turn restores LDHA expression and ultimately recovers glycolysis and cell proliferation. This model explains how CA IX-mediated pHi control allows for maintenance of glycolytic flux as a key part of metabolic reprogramming of cancer cells in hypoxia (Figure 4F).

## Discussion

Tumor hypoxia and acidosis represent toxic microenvironmental factors that can select for most aggressive cancer cells able to adapt to low oxygen and reinforce pH control (25). Proper pH is critically important for survival and behavior of cancer cells. While acidosis resulting from extracellular accumulation of metabolic products (such as lactate, protons, and carbon dioxide) enhances cancer cell invasion and metastasis, intracellular acidosis restricts biosynthetic reactions, hampers proliferation, causes catastrophic loss of ATP synthesis and ultimately leads to cell death (8, 26).

Hypoxia-induced carbonic anhydrase IX participates in shaping the tumor phenotype as a cell-surface component of both pH regulation machinery and cell migration-invasion apparatus that robustly support cancer cell survival and progression. Experimentally, genetic deletion or chemical inhibition of CA IX results in decreased model tumor formation (27, 28). CA IX acts as an extracellular pH-stat, maintaining an acidic tumor extracellular pH that is tolerated by cancer cells and results in a pro-metastatic behavior (29, 30). At the same time, CA IX stabilizes intracellular pH that is conducive to survival and proliferation (11, 21).

pH regulation mediated by CA IX is known to occur primarily via catalyzing a hydration of extracellular CO_2_ molecules to HCO_3_^−^ and H^+^ ions. HCO_3_^−^ ions are transported by bicarbonate transporters into the cell cytoplasm to alkalinize the pHi, whereas protons contribute to extracellular acidosis. This catalytic mechanism can successfully operate in acidic and lactate-rich tumor microenvironment typical for glycolytic tumor cells. CA IX is unique among CAs because its catalytic activity is insensitive to lactate concentrations up to 150 mM and extracellular lactate even stimulates its expression (31, 32). However, CA IX can regulate pH also via non-catalytic cooperation with monocarboxylate transporters through its proteoglycan-like domain serving as a collecting/distributing antenna for accelerated proton extrusion, which simultaneously augments efflux of lactate (33), stimulating proliferation in hypoxia.

Here we identify an additional mechanistic connection linking pH-regulating activity of CA IX to glycolysis. We show that CA IX is required for full expression and activity of a key glycolytic enzyme LDHA, while reduced expression or inactivation of CA IX in hypoxia-exposed cancer cells results in loss of control over intracellular pH, decreased glycolytic flux and reduced cell proliferation. Our finding that all three phenomena can be restored by treatment of CA IX-deficient cells with an alternative LDHA substrate α-KB further underlines their interdependence. Since α-KB does not exhibit any significant effect on pHi control of CA IX-expressing cells in normoxia or hypoxia, CA IX is apparently sufficient to optimize pHi regulation, glycolytic flux and consequently proliferation in a mutually coupled manner. Thus, all the available data suggest that CA IX stabilizes intracellular pH by several simultaneously occurring and interconnected mechanisms, namely by facilitating production/import of bicarbonate ions, accelerating production/extrusion of protons and by maximizing glycolytic flux through increased level/activity of LDHA.

In this intimately coupled scenario, it is difficult to dissect the hierarchy of the observed phenomena. In tumors, reinforced pH control allows for faster glycolytic flux and proliferation, which in turn increases demand for oxygen and metabolites. This relationship is evidenced by data from patients' tumor specimens derived from diverse tissue types, where CA IX is often correlated, co-expressed and/or spatially overlapped with the biomarkers of glycolytic metabolism (including MCT1, MCT4, and LDH5) and with glucose consumption rate determined by ^18^FDG-PET (34–36). These data underpin the existence of *in vivo* link between hypoxia, CA IX and glycolysis in patients.

In conclusion, we demonstrate that in addition to its role as a pH regulator and pro-metastatic factor, CA IX facilitates metabolic adaptation to hypoxia through supporting glycolysis and cellular proliferation. Because CA IX is expressed almost exclusively in tumors, this study provides additional insight how targeting CA IX could be an effective anticancer strategy, and suggests that highly glycolytic tumors might be particularly vulnerable to CA IX-directed agents.

## Data Availability Statement

The raw data supporting the conclusions of this article will be made available by the authors, without undue reservation.

## Author Contributions

MB performed most of the experiments and was a major contributor in writing the manuscript. ES contributed to the design of the study and interpretation of data. RB contributed to validation experiments. JK, MK, and AG generated cells and cell lines with CA IX suppression or knockout. MV participated in the proteomic experiments. NZ participated in the design of proteomic experiments and data interpretation. SA and AS performed the MS analysis and protein identification. IP participated in Seahorse experiments. ND supervised the study, contributed to design and was a major contributor in writing the manuscript. SP designed and supervised the study and was a major contributor in writing the manuscript. All authors contributed to the article and approved the submitted version.

## Conflict of Interest

SP is a co-inventor of patents related to CA IX. The remaining authors declare that the research was conducted in the absence of any commercial or financial relationships that could be construed as a potential conflict of interest.
