# Supplementary material for: CA IX Stabilizes Intracellular pH to Maintain Metabolic Reprogramming and Proliferation in Hypoxia
Source: Front Oncol. 2020 Sep 2;10:1462. doi: 10.3389/fonc.2020.01462 (PMC7493625; doi:10.3389/fonc.2020.01462)
Supplement: Supplementary file 1 [file Table_1.DOCX]

**Supplementary Table 1.** **Proteins identified as differentially represented during proteomic analysis of sh-RNA mediated CA IX knockdown carcinoma cells vs control.** Cells were exposed to hypoxia as described in the experimental section before sampling for proteomic analysis. Spot number, fold change, protein name, accession number, entry and gene name, identification score, sequence coverage (%) and function are reported.

| **Down-represented after CA IX silencing** | | | | | | | | |  |
| --- | --- | --- | --- | --- | --- | --- | --- | --- | --- |
| **Spot** | **Av.**  **Ratio** | **Protein name** | **Accession No.** | **Entry name** | **Gene**  **name** | **Mascot  score** | **Coverage** | **Description and/or**  **function** | |
| 2132 | -2.22 | Profilin-1 | P07737 | PROF1 | PFN1 | 405 | 43.6 | Small actin-binding protein that plays an important role in actin dynamics by regulating actin polymerization in response to extracellular signals | |
| 1681 | -1.65 | Cathepsin B heavy chain | P07858 | CATB | CTSB | 117 | 10.3 | Lysosomal cysteine proteinase implicated in tumor invasion and metastasis | |
| 1108 | -1.62 | Phosphoglycerate kinase | P00558 | PGK1 | PGK1 | 1256 | 68.6 | Glycolytic enzyme that catalyzes conversion of 1,3-diphosphoglycerate to 3-phosphoglycerate | |
| 1153 | -1.34 |  |  |  |  | 1503 | 83.5 |  |  |
| 1404 | -1.61 | L-lactate dehydrogenase A chain | P00338 | LDHA | LDHA | 788 | 43.7 | Catalyzes conversion of L-lactate and NAD to pyruvate and NADH in the final step of anaerobic glycolysis | |
| 1401 | -1.45 |  |  |  |  | 820 | 50.9 |  |  |
| 800 | -1.46 | Prolyl 4-hydroxylase subunit alpha-1 | P13674 | P4HA1 | P4HA1 | 1139 | 41.9 | Subunit of a key enzyme in collagen synthesis, catalyzes post-translational formation of 4-hydroxyproline in -Xaa-Pro-Gly- sequences in collagens and other proteins | |
| 1102 | -1.59 | Alpha-enolase | P06733 | ENOA | ENO1 | 1407 | 53.2 | Glycolytic enzyme implicated in growth control and hypoxia tolerance. Catalyzes conversion of 2-phosphoglycerate to phosphoenolpyruvate. Spliced isoform binds c-myc promoter and functions as tumor suppressor | |
| 1550 | -1.52 | Triosephosphate isomerase | P60174 | TPIS | TPI1 | 443 | 39.5 | Catalyzes isomerization of glyceraldehydes 3-phosphate (G3P) and dihydroxy-acetone phosphate (DHAP) in glycolysis and gluconeogenesis | |
|  |  | Endoplasmic reticulum resident protein 29 | P30040 | ERP29 | ERP29 | 357 | 23.4 | Plays an important role in the processing of secretory proteins within the endoplasmic reticulum (ER), possibly in their folding process. | |
| 1618 | -1.37 | Triosephosphate isomerase | P60174 | TPIS | TPI1 | 914 | 68.2 | Catalyzes isomerization of glyceraldehydes 3-phosphate (G3P) and dihydroxy-acetone phosphate (DHAP) in glycolysis and gluconeogenesis | |
| 2327 | -1.52 | GTP cyclohydrolase 1 feedback regulatory protein | P30047 | GFRP | GCHFR | 176 | 60.7 | Implicated in phenylalanine metabolism in the liver and in the production of biogenic amine neurotransmitters and nitric oxide | |
| 1342 | -1.47 | Annexin A2 | P07355 | ANXA2 | ANXA2 | 1183 | 65.2 | Calcium-dependent phospholipid-binding protein involved in regulation of cellular growth and in signal transduction pathways | |
| 1352 | -1.46 |  |  |  |  | 1029 | 58.4 |  |  |
| 1217 | -1.45 | Annexin A2 | P07355 | ANXA2 | ANXA2 | 629 | 41.9 | Calcium-dependent phospholipid-binding protein involved in regulation of cellular growth and in signal transduction pathways | |
|  |  | Fructose-biphophate aldolase A | P04075 | ALDOA | ALDOA | 709 | 44.0 | Glycolytic enzyme that catalyzes reversible conversion of fructose-1,6-bisphosphate to glyceraldehyde 3-phosphate and dihydroxyacetone phosphate | |
| 1234 | -1.41 | Fructose-biphophate aldolase A | P04075 | ALDOA | ALDOA | 858 | 50.3 | Glycolytic enzyme that catalyzes reversible conversion of fructose-1,6-bisphosphate to glyceraldehyde 3-phosphate and dihydroxyacetone phosphate | |
| 1546 | -1.46 | Phosphoglycerate mutase 1 | P18669 | PGAM1 | PGAM1 | 530 | 50.0 | Catalyzes reversible reaction of 3-phosphoglycerate (3-PGA) to 2-phosphoglycerate (2-PGA) in the glycolytic pathway | |
| 735 | -1.44 | Phosphoacetylglucosamine mutase | O95394 | AGM1 | PGM3 | 645 | 29.5 | Mediates both glycogen formation and utilization by catalyzing the interconversion of glucose-1-phosphate and glucose-6-phosphate | |
| 1695 | -1.43 | Peroxiredoxin-6 | P30041 | PRDX6 | PRDX6 | 358 | 37.9 | Thiol-specific antioxidant protein, involved in regulation of phospholipid turnover as well as in protection against oxidative injury. Activates JAK/STAT3 pathway | |
| 1169 | -1.4 | 60S acidic ribosomal protein P0 | P05388 | RLA0 | RPLP0 | 586 | 43.8 | RNA binding and structural component of the 60S ribosomal subunit. | |
| 833 | -1.38 | 60 kDa heat shock protein | P10809 | CH60 | HSPD1 | 1780 | 51.0 | Mitochondrial protein, essential for the folding and assembly of newly imported proteins in the mitochondria | |

| **Over-represented after CA IX silencing** | | | | | | | | |
| --- | --- | --- | --- | --- | --- | --- | --- | --- |
| **Spot** | **Av.**  **Ratio** | **Protein name** | **Accession No.** | **Entry name** | **Gene**  **name** | **Mascot  score** | **Coverage** | **Description and/or**  **function** |
| 1764 | 1.41 | Superoxide dismutase [Mn] | P04179 | SODM | SOD2 | 193 | 22.5 | Mitochondrial protein that binds to the superoxide byproducts of oxidative phosphorylation and converts them to hydrogen peroxide and diatomic oxygen |
| 1107 | 1.45 | Cytochrome b-c1 complex subunit 1 | P31930 | QCR1 | UQCRC1 | 867 | 41.5 | Component of the mitochondrial respiratory chain (Complex III), may mediate formation of the complex between cytochromes b and c1 |
| 723 | 1.46 | Heat shock cognate 71 kDa protein | P11142 | HSPA8 | HSPA8 | 656 | 21.7 | Molecular chaperone implicated in protection of the proteome from stress, folding/transport of novel polypeptides, and activation of proteolysis of misfolded proteins. Inhibits the transcriptional coactivator activity of CITED1 on Smad-mediated transcription. Component of the PRP19-CDC5L complex forming an integral part of the spliceosome and required for pre-mRNA splicing |
|  |  | Stress-70 protein | P38646 | GRP75 | HSPA9 | 735 | 26.4 | Localized in the mitochondria but also in the endoplasmic reticulum, plasma membrane and cytoplasmic vesicles. Negative regulator of RAF/MEK/ERK pathway, tumor suppressor (known as mortalin) |
| 683 | 1.85 | Stress-70 protein | P38646 | GRP75 | HSPA9 | 1350 | 40.1 | Localized in the mitochondria but also in the endoplasmic reticulum, plasma membrane and cytoplasmic vesicles. Negative regulator of RAF/MEK/ERK pathway, tumor suppressor (known as mortalin) |
| 599 | 1.46 | Stress-70 protein | P38646 | GRP75 | HSPA9 | 1003 | 38.1 | Localized in the mitochondria but also in the endoplasmic reticulum, plasma membrane and cytoplasmic vesicles. Negative regulator of RAF/MEK/ERK pathway, tumor suppressor (known as mortalin) |
|  |  | Glycine-tRNA ligase | P41250 | SYG | GARS | 913 | 31.1 | Primarily localized to the mitochondria, but also found in the endoplasmic reticulum, plasma membrane and cytoplasmic vesicles. Implicated in the control of cell proliferation and cellular aging |
| 592 | 1.52 | Glycine-tRNA ligase | P41250 | SYG | GARS | 922 | 29.1 | Primarily localized to the mitochondria, but also found in the endoplasmic reticulum, plasma membrane and cytoplasmic vesicles. Implicated in the control of cell proliferation and cellular aging |
| 1370 | 1.50 | Annexin A5 | P08758 | ANXA5 | ANXA5 | 821 | 56.3 | Annexin 5 is a phospholipase A2 and protein kinase C inhibitory protein with calcium channel activity and a potential role in cellular signal transduction, inflammation, growth and differentiation |
| 1309 | 1.53 | Serine/threonine-protein phosphatase PP1-beta catalytic subunit | P62140 | PPP1CB | PPP1CB | 554 | 39.4 | Protein phosphatase that associates with over 200 regulatory proteins to form highly specific holoenzymes which dephosphorylate hundreds of biological targets. Protein phosphatase (PP1) is essential for cell division, it participates in the regulation of glycogen metabolism, muscle contractility and protein synthesis. |
| 1061 | 1.60 | Eukaryotic initiation factor 4A-1 | P60842 | IF4A1 | EIF4A1 | 1055 | 54.9 | ATP-dependent RNA helicase that is a subunit of the eIF4F complex involved in cap recognition and is required for mRNA binding to ribosome |
| 2140 | 1.62 | Profilin-1 | P07737 | PROF1 | PFN1 | 487 | 67.1 | Small actin-binding protein, plays an important role in actin dynamics by regulating actin polymerization in response to extracellular signals |
| 971 | 1.66 | Heterogenous nuclear ribonucleoprotein H | P31943 | HNRH1 | HNRNPH1 | 587 | 30.5 | Associates with pre-mRNAs in the nucleus and influences pre-mRNA processing and other aspects of mRNA metabolism and transport |
| 1116 | 1.68 | Protein NDRG1 (N-Myc downstream regulated 1) | Q92597 | NDRG1 | NDRG1 | 486 | 29.2 | Cytoplasmic protein involved in stress responses, hormone responses, cell growth, and differentiation, necessary for p53-mediated caspase activation and apoptosis. Metastasis suppressor |
| 1627 | 1.70 | Rho GDP-dissociation inhibitor 1 | P52565 | GDIR1 | ARHDGIA | 169 | 22.1 | Inhibits the disassociation of Rho family members from GDP (guanine diphosphate), thereby maintaining these factors in an inactive state |
| 953 | 1.79 | Histidine-tRNA ligase | P12081 | SYHC | HARS | 653 | 23.6 | Responsible for the synthesis of histidyl-transfer RNA, which is essential for the incorporation of histidine into proteins |
