# Supplementary material for: CA IX Stabilizes Intracellular pH to Maintain Metabolic Reprogramming and Proliferation in Hypoxia
Source: Front Oncol. 2020 Sep 2;10:1462. doi: 10.3389/fonc.2020.01462 (PMC7493625; doi:10.3389/fonc.2020.01462)
Supplement: Supplementary file 2 [file Data_Sheet_1.pdf]

Supplementary Material

1 Supplementary Material and Methods

Cell models

Cell line	Modification	Type/Description	Reference
HeLa	Parental and scrambled controls	Cervical cancer, tumorigenic, CA9 inducible by hypoxia	RRID:CVCL_0030
HeLa DOX	Stable doxycycline-inducible CA IX knockdown using shRNA		(14)
HeLa KD	Transient CA IX knockdown using a pool of CA9 siRNAs		See below
HeLa dCA	Stable transfectants expressing CA IX with deletion of catalytic domain		(12)
HT-1080	Parental and scrambled controls	Fibrosarcoma, heterozygous for IHD1 mutation, tumorigenic, CA9 inducible by hypoxia	RRID:CVCL_0317
HT-1080 KO	Stable HT-1080 CA IX knockout generated using CRISPR/Cas9-mediated genome editing		See below
HT-1080 KD	Transient CA IX knockdown using a pool of CA9 siRNAs		See below
A-549	Parental and scrambled controls	Lung cancer, tumorigenic, CA9 inducible by hypoxia	RRID:CVCL_0023
A-549 KD	Transient CA IX knockdown using a pool of CA9 siRNAs		See below
C-33A	Parental and mock control	Cervical Cancer, non-tumorigenic, no endogenous CA IX expression	RRID:CVCL_1094
C-33A FL	Stable transfectants constitutively expressing full-length CA IX		(12)

Preparation of transient HT-1080 KD, HeLa KD and A-549 KD cells

HT-1080, HeLa and A-549 cells with transient CA IX knockdown (HeLa KD and A-549 KD) we generated using ON-TARGETplus siRNA pool (Dharmacon) of 4 CA9-targeting siRNA sequences and 4 control siRNA sequences as follows:

ON-TARGET CA9 siRNA 1: UACCUGAAGUUAAGCCUAA

ON-TARGET CA9 siRNA 2: CAGCUGAACUUCCGAGCGA

ON-TARGET CA9 siRNA 3: GGACAUAUCUGCACUCCUG

ON-TARGET CA9 siRNA 4: AGGAGGAUCUGCCCAGUGA

ON-TARGET CTRL siRNA 1: UGGUUUACAUGUCGACUAA

ON-TARGET CTRL siRNA 2: UGGUUUACAUGUUGUGUGA

ON-TARGET CTRL siRNA 3: UGGUUUACAUGUUUUCUGA

ON-TARGET CTRL siRNA 4: UGGUUUACAUGUUUUCCUA

Preparation of stable HT-1080 clones

HT-1080 KO cells were prepared by CRISPR/Cas9-mediated genome editing using double nickase strategy. Cells were stably co-transfected by a pair of vectors expressing two adjacent gRNAs targeting promoter region of CA9 gene (for sequences see Supplementary Information) and mutated nuclease (nickase). Deletions of target sequences in selected clones were sequence-verified on genomic level.

Vector-A: **pX335-dHRE-A** with cloned target sequence: (5´ → 3´) GCCCGTACACACCGTGTGCT (-42 → -22 from ATG)

Vector-B: **pX335-dHRE-B** with cloned target sequence: (5´ → 3´) GTGCATTGGAAACGAGAGCT (-67 → -42 from ATG)

pX335-U6-Chimeric_BB-CBh-hSpCas9n(D10A) (Addgene #42335)

2D proteomic profiling

Sample preparation. $1.5x10^6$ HeLa DOXCA IX shRNA cells were incubated for 48 h in DMEM media containing 1 mM dimethyloxalyglycine (DMOG) with concurrent serum starvation (0.5% fetal calf serum). shRNA-mediated CA IX KD was induced by addition of 250 pg doxycycline (DOX) into growth media 72 h prior to seeding and maintained throughout the whole experiment. Three biological samples of CA IX KD and control cells were prepared. Cell lysates were obtained in 1% v/v Triton X-100, 0.1% w/v SDS, 150 mM NaCl, 1 M Tris, pH 7.5. Protein concentration values were determined using the BCA Protein assay kit (Thermo Scientific). Proteins were precipitated overnight at -20 °C by

adding 9 vol of acetone:methanol 9:1 v/v, and recovered by centrifugation at 8,500 rpm for 30 min, at 4 °C. Obtained pellets were air-dried and resuspended in 7 M urea, 2 M thiourea, and 4% CHAPS. Final protein concentration values were determined using the Bradford method.

Sample labeling and 2D DIGE analysis. The pH value of each sample was adjusted to pH 8.5 with Tris-HCl. Fifty µg of proteins belonging to each sample were labeled with 400 pmol of Cy2-, Cy3- or Cy5-dyes (GE Healthcare), using a dye-swapping strategy. A mixture of the 6 samples was labeled with Cy2 dye, as the internal standard required by the 2D-DIGE protocol. Each labeling reaction was performed in the dark, for 30 min at 0 °C, and quenched with 1 mM lysine. Appropriate Cy3- and Cy5-labeled pairs and a Cy2-labeled control were used to generate mixtures. Each mixture was supplemented with 1% v/v IPG buffer, pH 3-10 NL (GE Healthcare), 1.4% v/v DeStreak reagent (GE Healthcare) and 0.2% w/v DTT to reach a final volume of 450 µl in 7 M urea, 2 M thiourea, and 4% w/v CHAPS. The mixtures were used for passive hydration of immobilized pH gradient IPG gel strips (24 cm, pH 3-10 NL) in the dark, for 16 h, at 20 °C. Isoelectric focusing (IEF) was carried out with an IPGphor II apparatus (GE Healthcare) up to 80,000 V/h at 20 °C. After IEF, each strip was equilibrated with an equilibration solution composed of 6 M urea, 2% w/v SDS, 20% w/v glycerol, and 0.375 M Tris–HCl (pH 8.8), in the presence of 0.5% w/v DTT, for 15 min, in the dark; then, it was equilibrated in the same buffer containing 4.5% w/v iodoacetamide, for another 15 min. Equilibrated IPG strips were transferred onto 12% polyacrylamide gels to perform the second-dimension SDS-PAGE, using an ETTAN DALT six electrophoresis system (GE Healthcare). Gels were scanned with a Typhoon 9400 variable mode imager (GE Healthcare) using proper excitation/emission wavelengths for Cy2 (488/520 nm), Cy3 (532/580 nm), and Cy5 (633/670 nm). Gel images were visualized with the Image-Quant software (GE Healthcare) and analyzed using the DeCyder 6.0 software (GE Healthcare). DeCyder differential In-gel-Analysis (DIA) module was used for spot detection and pairwise comparison of each sample (Cy3 and Cy5) to the Cy2 mixed standard present in each gel. Then, the DeCyder Biological Variation Analysis (BVA) module was used to simultaneously match all of the protein-spot maps from the gels, and to calculate average abundance ratios and statistical parameters (Student's T-test). Differentially represented spots were identified as those having a relative representation ratio > 1.38 or < 1.38, with a P value ≤ 0.05.

Preparative 2D Gel. Preparative two-dimensional gel electrophoresis (2-DE) was performed using 500 µg of mixed unlabeled protein samples. It was performed as reported above, without previous sample labelling. The resulting 2-DE gel was stained with Sypro Ruby protein gel stain (Thermo Fisher Scientific, USA). After spot matching with the master gel from the analytical assay in the BVA module of DeCyder software, a pick list was generated for spot picking by a robotic picker (Ettan spot picker, GE Healthcare, UK).

Mass spectrometry analysis for protein identification. Gel spots were triturated, washed with water, in-gel reduced with DTT, S-alkylated with iodoacetamide, and then digested with trypsin [1]. Resulting peptide mixtures were desalted by µZip-TipC18 (Millipore) using 50% v/v acetonitrile, 5% v/v formic acid as eluent. Recovered peptides were then analyzed for protein identification by nanoLC-ESI-LIT-MS/MS, using an LTQ XL mass spectrometer (Thermo Fisher Scientific, USA) equipped with a Proxeon nanospray source connected to an Easy-nanoLC (Proxeon, Denmark) [2]. Peptides were resolved on an Easy C18 column (100 mm × 0.075 mm, 3 µm) (Proxeon); mobile phases were 0.1% v/v formic acid and 0.1% v/v formic acid in acetonitrile, running at a total flow rate of 300 nL/min. Spectra were acquired in the range *m/z* 400−2000. Each peptide mixture was analyzed under collision-induced dissociation-MS/MS data-dependent product ion scanning procedure, enabling dynamic exclusion

(repeat count 1 and exclusion duration 60 sec), over the 3 most abundant ions. Mass isolation window and collision energy were set to *m/z* 3 and 35%, respectively.

Raw data from nLC-ESI-LIT-MS/MS analysis were searched by MASCOT search engine (version 2.2.06, Matrix Science, UK) against an updated (2017/03) UniProtKB non-redundant human database in order to identify proteins from gel spots. Database searching was performed by using Cys carbamidomethylation and Met oxidation as fixed and variable protein modifications, respectively, a mass tolerance value of 1.8 Da for the precursor ion and 0.8 Da for MS/MS fragments, trypsin as proteolytic enzyme, and a missed cleavage maximum value of 2. Other MASCOT parameters were kept as default. Protein candidates assigned on the basis of at least two sequenced peptides with an individual peptide expectation value < 0.05 (corresponding to a confidence level for peptide identification > 95%) were considered confidently identified. Definitive peptide assignment was always associated with manual spectra visualization and verification. Protein identity was definitively assigned only when an emPAI1st to emPAI2nd ratio >2 was observed [3].

1. Scippa, G.S., *et al.* The proteome of lentil (Lens culinaris Medik.) seeds: discriminating between landraces. *Electrophoresis* **31**, 497-506 (2010).

2. Buanne, P., *et al.* Characterization of carbonic anhydrase IX interactome reveals proteins assisting its nuclear localization in hypoxic cells. *Journal of proteome research* **12**, 282-292 (2013).

3. Shinoda, K., Tomita, M. & Ishihama, Y. emPAI Calc--for the estimation of protein abundance from large-scale identification data by liquid chromatography-tandem mass spectrometry. *Bioinformatics (Oxford, England)* **26**, 576-577 (2010).

2 Supplementary Figures and legends

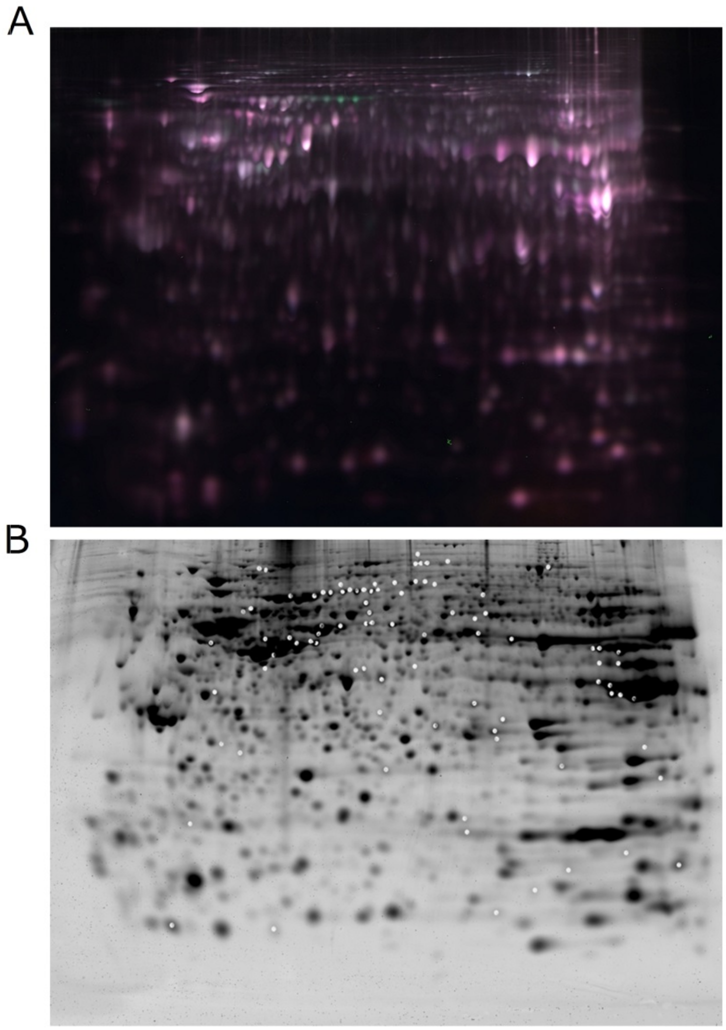

Supp. Figure S1. (**A**) Representative 2D-DIGE pre-stained analytical gel (n=3) showing the effect of CA IX knockdown on the proteome of HeLa DOX cells treated with 1mM DMOG for 48 hours. (**B**) SYPRO Ruby stained preparative gel with picked proteins of interest.

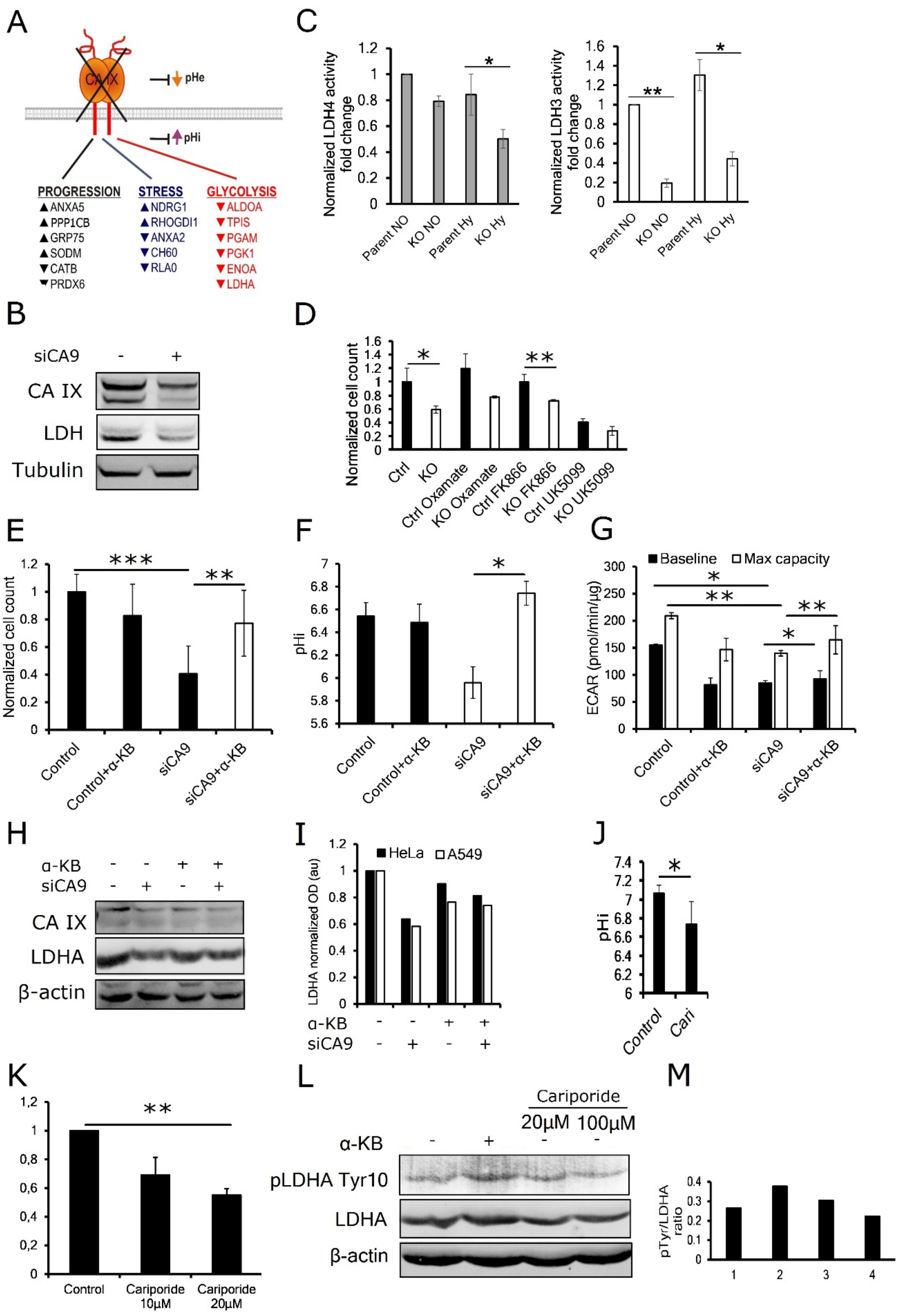
A
CA IX
pHe
pHi
PROGRESSION
ANXA5
PPP1CB
GRP75
SODM
CATB
PRDX6
STRESS
NDRG1
RHOGDI1
ANXA2
CH60
RLA0
GLYCOLYSIS
ALDOA
TPIS
PGAM
PGK1
ENOA
LDHA
B
siCA9 - +
CA IX
LDH
Tubulin
C
Normalized LDH4 activity fold change
Parent NO
KO NO
Parent Hy
KO Hy
Normalized LDH3 activity fold change
D
Normalized cell count
Ctrl
KO
Ctrl Oxamate
KO Oxamate
Ctrl FK866
KO FK866
Ctrl UK5099
KO UK5099
E
Normalized cell count
Control
Control+α-KB
siCA9
siCA9+α-KB
F
pHi
G
Baseline
Max capacity
ECAR (pmol/min/µg)
H
α-KB - - + +
siCA9 - + - +
CA IX
LDHA
β-actin
I
HeLa
A549
LDHA normalized OD (au)
α-KB - - + +
siCA9 - + - +
J
pHi
Control
Cari
K
Control
Cariporide 10µM
Cariporide 20µM
L
Cariporide
20µM 100µM
α-KB - + - -
pLDHA Tyr10
LDHA
β-actin
M
pTyr/LDHA ratio
1 2 3 4

Supp. Figure S2. (**A**) Schematic representation of the effects of CA IX loss on the HeLa proteome. (**B**) Representative immunoblot showing the effect of CA IX knockdown in HT1080 KD cells treated with 1% O_2 for 48 hours. (**C**) LDH4 (left) and LDH3 (right) activity of HT-1080 cells in response to α-KB in normoxia (n=3). (**D**) Cell number of HT-1080 KO cells incubated for 72 hours in 1% O_2 in the presence of 50 mmol/l oxamate, 10 nmol/l FK866 or 10 µmol/l UK5099. (**E**) Cell number of A-549 KD cells after 72 hours in 1% O_2 in the presence or absence of α-KB. (**F**) pHi of A-549 KD cells incubated for 48 hours in 1% O_2 with 1 mM α-KB using SNARF-1 pH indicator. (**G**) Seahorse analysis of baseline ECAR (black) and the maximal glycolytic capacity (white) of A-549 KD cells. (**H**) Representative immunoblot showing LDHA levels in control and α-KB-treated HT-1080 KD cells incubated for 48 hours in 1% O_2. (**I**) LDHA levels in control and α-KB-treated A-549 KD cells incubated for 48 hours in 1% O_2 quantified relative to β-actin in HeLa and A-549 cells. (**J**) pHi measurements of HT-1080 cells incubated with 20 µM cariporide in growth medium with 10 mmol/l PIPES buffer (pKa=6.72) for 24 hours in normoxia. (**K**) Densitometry analysis of LDHA levels in HT1080 cells treated with cariporide for 24 hours in normoxia (n=3). (**L**) Immunoblot showing the effect of 48h cariporide and α-KB treatment on the levels of activating LDHA tyrosine 10 phosphorylation (pLDHA Tyr10) in normoxic A-549 cells. P values were calculated against Control by t test. $^{*}P < 0.05$; $^{**}P < 0.01$
